# Biological Activities of Paeonol in Cardiovascular Diseases: A Review

**DOI:** 10.3390/molecules26164976

**Published:** 2021-08-17

**Authors:** Shalini Vellasamy, Dharmani Murugan, Razif Abas, Aspalilah Alias, Wu Yuan Seng, Choy Ker Woon

**Affiliations:** 1 Department of Microbiology and Parasitology, School of Medicine, Faculty of Medicine, Bioscience and Nursing, MAHSA University, Jenjarum 42610, Selangor, Malaysia; shalini.v@mahsa.edu.my; 2 Department of Pharmacology, Faculty of Medicine, University of Malaya, Kuala Lumpur 50603, Malaysia; dharmani79@um.edu.my; 3 Department of Human Anatomy, Faculty of Medicine and Health Sciences, Universiti Putra Malaysia, Seri Kembangan 43400, Selangor, Malaysia; razifabas@upm.edu.my; 4 Department of Basic Sciences and Oral Biology, Faculty of Dentistry, Universiti Sains Islam Malaysia, Kuala Lumpur 55100, Malaysia; draspa76@usim.edu.my; 5 Fakultas Kedokteran Gigi, Faculty of Dental Medicine, Universitas Airlangga, Surabaya 60132, Indonesia; 6 Centre for Virus and Vaccine Research, Sunway University, Bandar Sunway 47500, Selangor, Malaysia; sengwu_21@yahoo.com; 7 Department of Biological Sciences, Sunway University, Bandar Sunway 47500, Selangor, Malaysia; 8 Department of Anatomy, Faculty of Medicine, Universiti Teknologi MARA, Sungai Buloh 47000, Selangor, Malaysia

**Keywords:** paeonol, cardiovascular diseases, anti-inflammatory, anti-oxidant, anti-apoptotic, vascular tone regulation

## Abstract

Paeonol is a naturally existing bioactive compound found in the root bark of *Paeonia suffruticosa* and it is traditionally used in Chinese medicine for the prevention and management of cardiovascular diseases. To date, a great deal of studies has been reported on the pharmacological effects of paeonol and its mechanisms of action in various diseases and conditions. In this review, the underlying mechanism of action of paeonol in cardiovascular disease has been elucidated. Recent studies have revealed that paeonol treatment improved endothelium injury, demoted inflammation, ameliorated oxidative stress, suppressed vascular smooth muscle cell proliferation, and repressed platelet activation. Paeonol has been reported to effectively protect the cardiovascular system either employed alone or in combination with other traditional medicines, thus, signifying it could be a hypothetically alternative or complementary atherosclerosis treatment. This review summarizes the biological and pharmacological activities of paeonol in the treatment of cardiovascular diseases and its associated underlying mechanisms for a better insight for future clinical practices.

## 1. Introduction

Cardiovascular disease (CVD) is one of the major causes of death in the world [1]. The World Health Organization estimates that CVD is responsible for the deaths of approximately 30,000 people each day and accounted for 15.2 million deaths worldwide in 2016 [2,3]. The United Nations in 2011, recognized non-communicable diseases, including CVD as a major concern for global health and dramatically planned to reduce the effect of this disease in all regions [4]. The global awareness was expanded with increased efforts to reduce CVD and other non-communicable diseases [5,6].

The mechanism of CVD from the molecular perspective has been studied extensively for the past decades. Oxidative stress and inflammation were found to be predominantly associated with the pathogenesis of CVD. The inflammation could have been triggered by oxidative stress which results from the excessive generation of reactive oxygen species/reactive nitrogen species (ROS/RNS). This contributes to low-density lipoprotein (LDL) oxidation, endothelial dysfunction or apoptosis, atherosclerotic plaque formation, plaque rupture, vascular remodeling, and atherothrombosis [7,8,9].

Natural products offer unique structural and chemical diversity that serves as a source of novel drug and therapeutic agents [10]. Paeonol is a phenolic compound mainly isolated from the root bark and/or cortex of genus Paeonia, predominantly found in *Paeonia* suffruticosa Andrew (Cortex. Moutan) compared to *Paeonia ostia* T. Hong and J. X. Zhang, *Paeonia clusii subsp. clusii* F.C. Stearn, *Paeonia mascula subsp. hellenica* Tzanoud, *Paeonia parnassica* Tzanoud, *Paeonia lactiflora* Pall, and *Paeonia broteri* Boiss and Reut [11,12]. A number of studies revealed that paeonol possesses many physiological activities, including vascular dilation [13], inhibit platelet aggregation, [14] formation of free radicals [15], and prevent cardiovascular diseases [16,17]. In recent years, a great number of studies on the pharmacological effects of paeonol and its mechanism of action have been reported. This article reviews the mechanism of action of paeonol in cardiovascular diseases particularly looking into the anti-oxidant, anti-inflammatory, anti-apoptotic, and its regulation of vascular tone.

## 2. Pharmacological Features of Paeonol

### 2.1. Bioactive Compounds in Cortex Moutan

**Figure 1:**
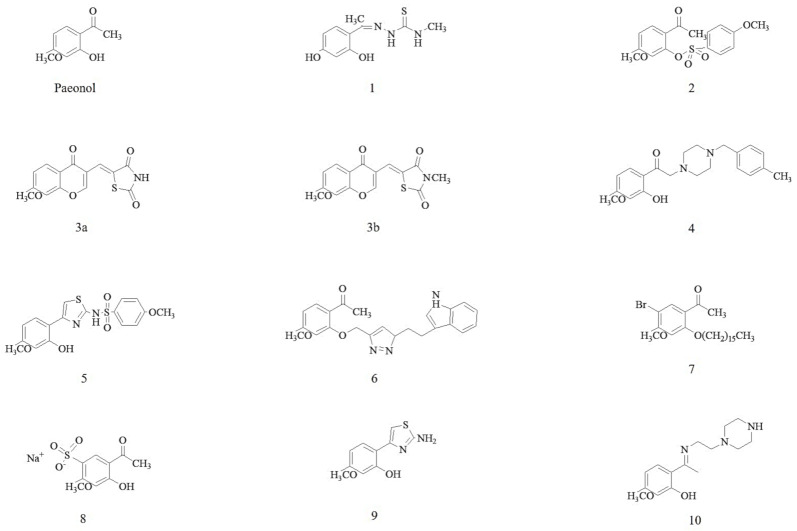
Chemical structures of paeonol and its derivatives (**1**–**10**). The derivatives are formed with thiosemicarbazone-like analogs (**1**), phenylsulfonyl-like analogs (**2**), chromonylthiazolidines-like analogs (**3a** and **3b**), donepezil-like analogs (**4**), aminothiazole-like analogs (**5**), tryptamine hybrid analogs (**6**), alkyl ether analogs (**7**), paeononlsilatie sodium (**8**), aminothiazole-like analogs (**9**), and piperazin-like analogs (**10**).

Several phytochemical studies performed on Cortex Moutan had identified and isolated approximately 80 bioactive compounds. The chemical structure of paeonol is as shown in Figure 1. Based on the structural formula, these bioactive compounds are divided into monoterpenoid glycosides, flavonoids, tannins, phenols, and paeonols. Paeonol (2′-hydroxy-4′-methoxyacetophenone) appeared to be the main representative bioactive compound in paeonol [18,19] and till today, twelve derivatives of paeonol have been synthesized (Figure 1). Among bioactive compounds derived from Cortex Moutan, paeonol has been extensively investigated for anti-cardiovascular activity and its mechanisms of action in CVD [20,21,22,23,24,25,26,27,28,29,30].

### 2.2. Pharmacokinetics and Drug Delivery of Paeonol

In the aspect of absorption, paeonol has been studied in different parameters, including, pH, drug concentration, osmotic pressure, and perfusion. Based on the results, it was noted that paeonol is absorbed by the intestinal tract and rapidly enters portal circulation upon oral administration [11,18]. Due to having a short half-life and maximum time, it is delivered rapidly in the bloodstreams to several target organs, including heart, liver, kidney, and brain without long-term accumulation and excreted from the body in a short time, conferring a great safety [31]. It has been reported that paeonol absorption is the first-order metabolism as it does not depend on its concentration. It can be absorbed easily in acidic conditions, particularly hypertonic solutions. Its absorption rate is constant similarly to its perfusion rate [32]. Chen et al. reported that experimental rats could absorb paeonol rapidly and efficiently via the intranasal route of administration [11,12]. Besides, the metabolism of paeonol has generated six metabolites as found in the urine samples of 19 Chinese volunteers who received 80 mg paeonol tablets for two days consecutively, three times per day. It has also shown that paeonol is metabolized by demethylation and hydroxylation in phase 1 metabolism followed by sulfuric acid and glucuronic acid conjugation in phase 2 metabolism [11,12].

Intriguingly, Li et al. revealed that the retention time and levels of paeonol in the heart and brain could be significantly increased via co-administration with other phytochemicals, as evidenced with danshensu in CVD [33,34]. Nonetheless, the therapeutic efficacy of paeonol was shown to be limited by the first-pass effect and poor bioavailability due to low aqueous solubility [18]. To counteract these issues, several nanotechnology-assisted drug delivery systems, for example, microemulsion gel, transethosome, porous microsphere, liquid crystalline nanoparticles, and microsponge, were developed, particularly to treat cancer and skin diseases [35,36,37,38,39,40]. These delivery systems had greatly enhanced the therapeutic effects of paeonol due to their high encapsulation capacity and stability, together with better control of drug release and retention time in the targeting tissues. Collectively, only a few drug systems that scheme for paeonol have been designed to test in cardiovascular diseases. For example, Chen et al. designed nanoemulsions for paeonol that effectively increase its protective effect in the cardiovascular system by enhancing its oral transport and absorption, and bioavailability due to evading p-glycoprotein-mediated drug efflux [41]. Besides, the better delivery of paeonol has been observed with its loading into stent microparticles or poly (butyl-2-cyanoacrylate) nanocapsules, resulting in greater prevention of restenosis and stent thrombosis after percutaneous coronary intervention [35,42]. Given the benefits of using nanotechnology-assisted drug delivery of paeonol, various nanoformulations should be designed and tested, with the aim of improving its therapeutic efficacy and prevention against CVD in clinical settings.

## 3. Mechanism of Action of Paeonol in Cardiovascular Diseases

Numerous studies have shown that paeonol has several therapeutic effects against cardiovascular diseases and protects the cardiovascular system via several mechanisms. 

### 3.1. Anti-Oxidant Mechanism

Exaggerated free radicals released by unopposed oxidative stress levels led to the increase in pathophysiology changes. Cardiovascular disease manifests major impact by this abnormality via mtDNA (mitochondrial DNA) damage, cellular apoptosis, and enzyme degradation [43]. MtDNA damage led to atherosclerosis via functional changes in the cell and disturbance of the mitochondrial respiratory chain [44]. Experimental and clinical studies have demonstrated endothelial dysfunction (ED) as an early predictor of cardiovascular pathology. ROS disturbs the function of endothelial nitric oxide synthase (eNOS) hence leading to impair nitric oxide (NO) bioavailability; a potent vasodilator, which contributes to essential hypertension [45]. Furthermore, ED manifested by lipid-rich atherosclerotic plaque rupture led to atherogenesis and myocardial infarction. This finding was proved by the development of oxidized low-density lipoproteins (ox-LDL) via ROS release. This bad lipid was shown to be oxidatively modified by vascular wall cellularity; endothelial cells, smooth muscle cells, and macrophages [46].

Oxidative stress occurs upon imbalance between the prooxidant-antioxidant condition in cells resulting in cellular apoptosis, hence promoting atherosclerosis [47]. An in vitro study using human umbilical vascular endothelial cells (HUVECs) showed 10 and 50 µM of paeonol significantly inhibited the overproduction of ROS when exposed by ox-LDL. A subsequent investigation by the same group revealed that the up-regulation of lectin-like low-density lipoprotein receptor-1 (LOX-1) protein expressions that were increased by ox-LDL were effectively suppressed by paeonol. This study further delineates the inhibition of ROS overproduction downregulated of p38 MAPK phosphorylation, NF-κB nuclear translocation, caspase-3 activation, and Bcl-2 production pathway, hence improved cell viability [48]. This result suggests that paeonol reduces ox-LDL in inducing apoptosis via ROS.

In atherosclerosis progression, vascular smooth muscle apoptosis or necrosis might result from cell senescence. The accumulation of its end products accumulates lipid peroxidation hence produce ROS [49]. Jamal et al. study highlighted the protective effect of paeonol in reducing the senescence effect in endothelial cells [50]. Pre-treatment with an optimum dose of 30 µM paeonol prior to H_2_O_2_ treatment normalized the pro-apoptotic p53 protein expression, as measured by fluorescent assay and western blotting. Downregulation of p53 protein suggests improvement of cellular senescence activity. Furthermore, paeonol increased Sirt1 expression, BrdU incorporation, and cell growth profile. Paeonol also reduced Ac-H3 K14 expression, Ac-H4 K16 expression, and SA-beta-galactosidase suggesting endothelial cells protection. This study suggests the relationship of paeonol in preventing aging-associated diseases, particularly, atherosclerosis and various cardiovascular disease [50].

Another study showed that paeonol was able to inhibit atherosclerotic plaque via downregulation of heme oxygenase-1 (HO-1) and upregulation of ATP-binding cassette transporter A1 (ABCA1) either via calpain activity reduction or wogonin increment in RAW264.7 macrophages and apolipoprotein E-deficient (ApoE^−/−^) mice. HO-1 mediates ABCA1, a cholesterol efflux regulatory protein to reduce the ox-LDL formation and inhibit foam cell formation. Treatment with paeonol, 5–50 µM decreased the expression of CD36 via inactivation of c-Jun-AP-1 pathway and increased the expression of ABCA1, hence reduced foam cells formation which further reduced intracellular lipid accumulation. Furthermore, the protein level of antioxidant enzymes, HO-1 in macrophages showed a dose-dependent increment in response to paeonol, thus demonstrating paeonol helps to regulate the atherosclerotic plaque formations via anti-oxidant properties [51].

### 3.2. Anti-Inflammatory Mechanism

Chronic inflammation leads to infiltration of inflammatory sensors such as tissue-resident macrophages, mast cells, and dendritic cells which induce the production of mediators including cytokines such as interleukins (ILs) and tumor necrosis factor-alpha (TNFα), chemokines, bioactive amines, and product of proteolytic cascades such as bradykinin. The overproduction of these mediators will eventually contribute to the progression of tissue damage [52]. The association between inflammation and cardiovascular disease, while seeming relatively recent, the root of this concept, stretches far back in time. Chronic inflammation is a common hallmark of cardiovascular diseases by accelerating atherosclerosis, destabilizing plaque, compromising endothelial functions, and causing arterial stiffness [53]. The inflammatory process leading to cardiovascular diseases includes an impaired function of endothelial cells, oxidative stress, accumulation of immune cells, production of TNFα, and several interleukins [54]. There have been studies relating inflammation as a risk factor in poor prognosis in ischemic damage as well as myocardial infarction [55]. Continuous generation of ROS by activated immune effector cells such as macrophages are also associated with chronic inflammatory conditions as the LDLs are oxidized by these ROS and then exert pro-atherogenic effects [56]. Activated endothelial cells then express adhesive molecules, such as vascular cell adhesion molecule-1 (VCAM-1), intercellular molecule 1 (ICAM-1), and selectins. These determine mononuclear cell recruitment into the vascular wall, together with the secretion of chemoattractant mediators such as complement factors, interleukin (IL)–8, and monocyte chemoattractant protein-1 (MCP-1). Monocytes differentiate into macrophages that become foam cells by ox-LDL absorption and then release a number of proinflammatory cytokines [57]. These inflammatory cascades impair the endothelial functions and the mechanical properties of the arteries. The decline in bioavailability of nitric oxide and the increase of the vasoactive peptides lead to arterial stiffening which in turn further causes endothelial dysfunction [53]. In the case of the inflammatory cascade in ischemic myocardial injury, cellular fragments will trigger the resident immune cells through the engagement of pattern recognition receptors (PRRs) for example membrane toll-like receptors, which activate inflammasomes in the heart, play a crucial role in cardiac remodeling. Excessive inflammation will induce cardiomyocyte apoptosis [55].

There have been reports documenting the anti-inflammatory properties of paeonol via various pathways and mechanisms. Pan et al. studied the effect of paeonol on TNF-α stimulated VCAM-1 expression of rat aortic endothelial cells (RAECs) [58]. In baseline conditions, VCAM-1 is not expressed but is rapidly induced in a pre-atherosclerotic state. Based on their investigation, it is shown that paeonol significantly inhibits TNF-α induced VCAM-1 expression as well as inhibits monocytic cell adhesion to TNF-α activated RAECs. Furthermore, their study also demonstrated the role of MAPK (ERK ½, JNK, and p38) in the process of VCAM-1 expression. MAPK was shown to block the activity of ERK ½, and p38. This evidence supports that paeonol reduces the monocytic cell adhesion to TNF-α induced RAECs by inhibiting the expression of VCAM-1 protein via the down-regulation of ERK ½ and p38. They found that paeonol was effectual in the prevention and treatment of atherosclerosis in the initial and progression stages through the inhibition of the inflammatory response [58].

There are also studies demonstrating paeonol exhibits anti-inflammatory effects through the suppression of TNFα, IL-1ß, inducible nitric oxide synthase (iNOS), cyclooxygenase-2 (COX-2), and prostaglandin E2 (PGE2) production in a rat model of carrageenan-evoked thermal hyperalgesia [59]. In models of hyperlipidemia, paeonol inhibited serum and aorta lipid peroxidation and decreased serum oxidative modification caused by ox-LDL in healthy people [18]. Kim et al. demonstrated via in vitro studies that paeonol significantly inhibited essential angiogenesis pathways such as proliferation and migration in fibroblast growth factor (FGF) stimulated HUVECs under pathological angiogenic conditions [59]. This finding indicates that paeonol is able to inhibit the early process of angiogenesis [59].

Another approach of paeonol as an anti-inflammatory is through the expression of miRNA-126 which has been reported specific to endothelial cells and also affects the pathophysiological processes of CVD such as atherosclerosis [60]. Yuan et al. conducted a study using ox-LDL stimulated vascular endothelial cells (VECs) isolated from rat thoracic aorta as an atherosclerosis model [60]. Ox-LDL significantly induced damage to VECs resulting in lower expression of miR-126, while treatment with paeonol promoted miR-126 expression to inhibit monocyte adhesion to ox-LDL-injured VECs via the downregulation of VCAM-1 expression. The same group demonstrated that paeonol blocks the activation of the PI3K/Akt/NF-κB signaling pathway which is a major pro-inflammatory signaling pathway in response to extracellular stress via promoting miR-126 expression. This finding suggests the possibility that miR-126 may serve as a therapeutic target along with paeonol as a treatment for atherosclerosis [60].

Another study by Liu et al. investigated the anti-inflammatory of paeonol via the expression of miRNA-21 [61]. Similar to the study by Yuan et al. ox-LDL stimulated VECs isolated from rat thoracic aortas were used as the study model [61]. The miR-21 may be critical in the progression of cardiovascular diseases as it is expressed abundantly in VECs, cardiomyocytes, and cardiac fibroblast. Based on the data obtained in this study, paeonol pre-treatment increased the survival rate of the ox-LDL stimulated VECs. The paeonol treatment also showed a significant decrease in ox-LDL-induced miR-21 expression as well as reversed the phosphatase and tensin homolog (PTEN) expression which is a downstream target gene for miR-21. Furthermore, paeonol decreased the ox-LDL induced TNF-α release. The transfection of miR-21 mimics or inhibitors combined with paeonol annulled or enhanced the TNF-α release respectively. Thus, there is a possibility the effect of paeonol on the release of TNF-α is miR-21 dependent [61].

Liu et al. carried out an in vivo and in vitro experiment to investigate the protective effect of paeonol on inflammatory response through the exosomal regulation of miR-223 [62]. High-fat diet ApoE^−/−^ mice were used to replicate the atherosclerosis model and HUVECs were used for the in vitro testing. There were several major findings from this research which include paeonol restricted the development of atherosclerosis lesion and inflammation by augmenting the miR-223 expression and inhibiting the STAT3 pathway on the aorta. Besides that, they also proved that paeonol, both in vivo and in vitro, could decline the expression levels of IL-1ß and IL6, cytokines that accelerate atherosclerosis and decrease VCAM-1 and ICAM-1. In addition, paeonol also enhanced the expression of miR-223 for both exosomes and co-cultured HUVECs which consequently inhibits the STAT3 pathway. The miR-223 is another possible target to be considered as a therapeutic target with the conjunction of paeonol [62].

Furthermore, there was an in-vivo study conducted by Ma et al. that investigated the potential of paeonol as a cardio-protective agent on the no-reflow phenomenon which is described as inadequate perfusion of the myocardium [63]. This is commonly caused by swelling of the endothelial tissue, leucocytes accumulation, and vasoconstriction which all lead to acute inflammation. The pre-treatment with paeonol reduced the no-reflow area in concurrence with a significant decline in the release of serum creatinine kinase (CK) and lactate dehydrogenase (LDH) as well as reduced troponin T (TnT) and C-reactive protein (CRP) levels, suggesting that paeonol pre-treatment decreased myocardial ischemic damage, most likely through modulation of the microvasculature. These demonstrate that paeonol can be used as an alternative in the treatment of myocardial no-reflow and other cardiovascular diseases [63].

In a model of ApoE^−/−^ mice, paeonol’s efficacy on cholesterol metabolism was investigated [64]. Paeonol significantly reduced the ox-LDL-induced cholesterol accumulation in macrophages due to cholesterol efflux. In addition, paeonol up-regulated the ATP-binding membrane cassette transport protein A1 (ABCA1) protein and mRNA but did not change the ABCG1 protein level. Paeonol effect on cholesterol efflux and cholesterol accumulation was inhibited by siRNA knockdown of liver X receptor α (LXRα). Paeonol stimulated the activity of nuclear translocation of LXRα. Furthermore, paeonol reduced atherosclerotic lesions, hyperlipidemia, and systemic inflammation as well as increased ABCA1 protein expression in aortas of paeonol-treated ApoE^−/−^ mice. These suggest that reduced the foam cells formation by stimulating LXRα-ABCA1–dependent cholesterol efflux in ApoE^−/−^ mice [64].

In summary, the active compound paeonol has considerable research evidence to be targeted as a therapeutic agent in atherosclerosis management which in consequence reduces the risk of CVD.

### 3.3. Regulation of Vascular Tone

The endothelium embodies a wide range of homeostatic functions fundamental for the regulation of vascular tone and structure by synthesizing and secreting a broad spectrum of substances including (a) endothelium-derived relaxing factors (EDRFs) [NO, prostacyclin and endothelium-derived hyperpolarizing factor (EDHF)], (b) endothelium-derived contracting factors (EDCF) [endothelin-1 and angiotensin II], (c) pro-inflammatory and pro-thrombolytic mediators and (d) growth factors [65]. NO is the primary EDRF released in most vascular beds and serves as a major endogenous local control of vascular tone. NO is formed from the conversion of a semi-essential amino acid, L-arginine to L-citrulline, by an enzyme called nitric oxide synthase (NOS) [66]. Once NO is released, it diffuses into smooth muscle cells of blood vessels and activates soluble guanylyl cyclase (sGC) to convert guanosine monophosphate (GMP) to cyclic GMP. cGMP is known to have a function in activating cGMP-dependent protein kinase through which it regulates several pathways involved in Ca^2+^ homeostasis, i.e., reduction in intracellular Ca^2+^ available for contraction and a decrease in the sensitivity of contractile proteins to Ca^2+^ which ultimately leads to vasodilatation [67].

Evidence from clinical and experimental studies has demonstrated that impairment of the endothelial function either initiates or is associated with the development and progression of cardiovascular diseases [68]. Endothelial dysfunction is characterized by a significant reduction in the bioavailability of vasodilator substances, in particular NO, and/or elevation in endothelium-derived contracting factors. This results in a dysfunctional endothelium, whereby it is the first step in the cascade of events leading to atherosclerosis and coronary diseases [69].

Numerous studies point to the loss of NO biological activity as a central mechanism of endothelial dysfunction. Endothelial dysfunction associated with eNOS uncoupling which leads to the production of ROS has been reported both in animal models such as angiotensin II-induced hypertension [70], streptozotocin (STZ)-induced diabetes [71], and hypertension-induced heart failure [72] as well as in patients with diabetes, hypertension, hypercholesterolemia, and atherosclerosis [73]. Besides eNOS uncoupling, enhanced EDCFs also increase the production of superoxide anion and cause cellular oxidative damage [74]. Disruption in the endothelium triggers a number of downstream signaling cascades that converge on the underlying vascular smooth muscle cells, thus disrupting the vasomotor function [75].

Studies have demonstrated that paeonol possesses a vasodilatory effect in the isolated aortic ring [13,76]. These studies have demonstrated the involvement of endothelium-dependent and independent mechanisms for its potent vasodilatory effect. The eNOS/NO was shown as the endothelium-dependent mechanism and inhibition of voltage-dependent and receptor-operated Ca^2+^ channel, as well as intracellular Ca^2+^ release as the endothelium-independent mechanism for its vasodilatory effect in rat aorta [13] and rat mesenteric artery [77].

In recent years, more studies have illustrated the protective role of paeonol in regulating vascular tone against dysfunction induced by exogenous stressors. In 2016, Choy et al. investigated the protective mechanism of paeonol against tunicamycin-induced endoplasmic reticulum stress in isolated mouse aortas. The study showed ex vivo treatment with 1 μM paeonol for 16 h reversed the impaired endothelium-dependent relaxations induced by tunicamycin in C57BJ/6J and PPARδ wild type (WT) mouse aortas. Moreover, the elevated ER stress markers, oxidative stress, and reduction of NO bioavailability induced by tunicamycin in both mouse aortas were reversed by paeonol treatment. However, these beneficial effects of paeonol were diminished in PPARδ knockout (KO) mouse aortas. Paeonol also was shown to increase the expression of 5’ adenosine monophosphate-activated protein kinase (AMPK) and PPARδ expression and activity while restoring the decreased phosphorylation of eNOS. Thus, paeonol protects against tunicamycin-induced vascular endothelial dysfunction by reducing endoplasmic reticulum stress-associated ROS levels and enhancing eNOS-induced NO production via the AMPK/PPARδ signaling pathway [78].

In 2017, the same group demonstrated the vascular protective effects of chronic treatment with paeonol on ER stress-induced endothelial dysfunction in mice [79]. Paeonol treatment of 20 mg/kg/day significantly ameliorated the impairment of endothelium-dependent relaxations of the aorta. Furthermore, paeonol reduced ROS levels in the mouse aorta and improved NO bioavailability in tunicamycin treated mice, thus further proving paeonol preserved endothelial function impaired by tunicamycin in vivo through the inhibition of ER stress-associated ROS.

Lipopolysaccharide (LPS) which is a major constituent of outer membranes of bacteria triggers inflammation, microvascular leaking, endothelial cells detachment, and eventually apoptosis leading to sepsis and its complication [80,81]. The protective effect of paeonol on vascular function was also demonstrated in endothelial dysfunction induced by lipopolysaccharides (LPSs) inflammatory conditions ex vivo and in vivo [82]. Ex vivo treatment with 1 μM paeonol reversed the impaired relaxation in response to the endothelium-dependent vasodilator acetylcholine in mouse aortae after exposure to LPS. Similarly, chronic co-treatment with 20 mg/kg/day paeonol reversed lipopolysaccharide-induced endothelial dysfunction. Furthermore, paeonol treatment downregulated the toll-like receptor 4 and bone-morphogenic protein 4 protein and its downstream signaling protein including iNOS and decreased ROS production which subsequently leads to an increase in NO bioavailability [82]. These studies concede that NO is an endothelial cell-protecting factor capable of suppressing apoptosis-related activities and elevating cellular survival.

In a recent study, paeonol was shown to decrease elevated blood pressure and increased cerebral blood flow velocity as well as decrease vascular endothelium injury in Spontaneously Hypertensive Rats [83]. They related the improvement in vascular function to the fact that paeonol reduced blood viscosity and oxidative stress and improved antioxidant capacity.

### 3.4. Anti-Apoptotic Mechanism

Apoptosis or programmed cell death occurs via activation of specific signaling pathways which requires a high level of energy leading to cell death [84]. Apoptosis plays a key role in multiple physiological processes such as embryogenesis, normal tissue homeostasis, and aging [85]. Recently, numerous reports in the literature have shown the involvement of apoptosis in the pathogenesis of various cardiovascular diseases. For instance, acute and chronic apoptosis of the myocytes occurs in acute myocardial infarction [86]. Animal and human studies showed that apoptosis occurs at the peri-infarcted region in the initial phase, proposing the role of apoptosis in acute myocardial loss after myocardial infarction [87]. Patients with heart failure are also associated with elevated rates of apoptosis which influence the infarction size and extend of left ventricle remodeling [88]. Apoptosis of endothelial cells triggers atherosclerosis while activated macrophages may kill the smooth muscle cells which disturb the stability of plaque [89].

Cardiovascular diseases can be induced via apoptotic mechanisms such as caspase-dependent or caspase-independent apoptosis [90]. In general, the caspase-dependent mechanisms can be subdivided into either extrinsic (involving death receptors) or intrinsic (mitochondria-mediated) pathways [91,92]. In the extrinsic apoptotic pathway, the binding of death ligands such as Fas ligand (FasL) or TNF-α to a membrane-bound death receptor initiates the death receptor-mediated pathway. This consequently activates caspase-8 followed by the downstream effector caspases [93]. Various literature reported that the extrinsic apoptotic pathway leads to the pathogenesis of heart failure [94,95,96]. In the intrinsic apoptotic pathway, the mitochondria are induced to release cytochrome c into the cytosol and form an activation complex called apoptotic protein activating factor-1 (Apaf-1) and caspase 9 [97,98]. Both extrinsic and intrinsic apoptotic pathways eventually merge into a common effector caspase called caspase-3 to complete the final biochemical and morphology apoptotic changes [99]. Meanwhile, the caspase-independent mechanism is related to the release of apoptotic factors including apoptosis-inducing factor (AIF) from mitochondria to the cytosol and subsequently nucleus translocation, leading to deoxyribonucleic acid (DNA) fragmentation without activation of caspase [100,101].

Cardiomyopathy induced by epirubicin, an antrum antibiotic remains a major worry as it led to significant side effects such as irreversible left ventricular dysfunction and congestive heart failure [102,103]. Wu et al. had discovered that paeonol protects against epirubicin-induced rat cardiac myocytes H9C2 and mice cardiomyocytes LH-1 apoptosis with a significant increase in viability rates and the number of apoptotic cells from the LTT assay and flow cytometry [104]. In TUNEL-stained mice heart sections, treatment with paeonol significantly reversed the apoptosis of cardiomyocytes induced by epirubicin from 78.19% to 36.83% [104]. Compared to the epirubicin-treated mice group, paeonol treated mice showed a decrease in cleaved caspase-3 and Bax as well as an increase in Bcl-2. Furthermore, paeonol-treatment reversed cardiac dysfunction induced by epirubicin which subsequently improved various physiological indexes such as reduction of left ventricular end-diastolic dimension (LVEDD) and left ventricular end-systolic diameter (LVESD), myocardial enzymes, inflammatory markers, and normalization of heart tissue morphology [104]. Paeonol also downregulated the protein expression of phosphoinositide 3-kinases (PI3K), protein kinase B (AKT), and mTOR in H9C2 and LH-1. These suggest that paeonol improved epirubicin-induced cardiomyopathy and cardiac dysfunction by reducing apoptosis via suppression of PI3K/AKT/mTOR signaling pathways [104]. In addition, in an arteriosclerotic in vivo model using ApoE^−/−^ mice, paeonol showed anti-atherosclerotic and anti-proliferation effects on vascular smooth muscle cells (VSMCs) by stimulating AMPK phosphorylation which reduced reduction p-mTOR/mTOR as well as induced conversion LC3II and therefore, inhibits the proliferation of cell in VSMCs [105].

Myocardial ischemia or reperfusion (I/R) injury can be caused by cellular apoptosis, leading to major cardiac injury [106,107]. The Notch1 signaling regulates the proliferation and differentiation of the cardiomyocytes in the heart [108,109]. In I/R-induced apoptosis in H9c2 embryonic rat myocardium-derived cells, pre-treatment with paeonol reduced the percentage of H9c2 cells apoptosis [110]. The anti-apoptotic effects of paeonol were associated with the downregulation of pro-apoptotic proteins such as cleaved-caspase-3 and Bax, as well as up-regulation of anti-apoptotic protein Bcl-2 [110]. In addition, paeonol up-regulated the Notch1 level which was reduced in I/R-induced apoptosis in H9c2, suggesting that paeonol protects SIR-induced H9c2 cells apoptosis via the Notch1 signaling pathway [110].

Ischemic heart disease (IHD) is a condition characterized by an imbalance between the demand of myocardial and oxygen supply from the coronary artery, leading to myocardium necrosis [111]. This results in various changes in pathophysiology and biochemistry, for instance, lipid peroxidation, hyperglycemia, or hyperlipidemia [111]. The protective effects and mechanism of paeonol were investigated in isoproterenol (ISO)-induced myocardial infarction in rats [112]. The results demonstrated that paeonol significantly suppressed ISO-induced apoptosis in the myocardial tissue as evidenced by a reduction in TUNEL-positive myocytes compared to the ISO-induced group in the myocardial tissue [112]. Paeonol treated group also showed reduced apoptosis signaling molecules namely Fas, TNF-α, Bax, caspase-3, caspase-8, caspase-9, cytochrome c while increased the anti-apoptotic protein such as Bcl-2 in myocardial tissue compared to the ISO-induced group. In addition, the paeonol-treated rat group showed lowered the myocardial injury markers level serum myocardial injury markers such as serum levels of serum creatine kinase-MB (CK-MB), cardiac troponin I (cTnI), and cardiac troponin T (cTnT), normalized morphological changes and mitigates lipid peroxidation in the myocardial heart tissue, suggesting that paeonol prevented myocardial injury induced by ISO [112]. During the exploration of underlying signaling pathways by paeonol, treatment with paeonol showed increased translocation of nuclear factor erythroid 2–related factor 2 (Nrf2) as well as enhanced phosphorylated PI3K and Akt which may lead to activation of its anti-oxidative and anti-apoptotic signaling in rats [112]. Collectively, the results suggest that the cardioprotective and anti-apoptotic effects of paeonol against ISO-induced myocardial infarction in rats were mediated via activation of Nrf2/PI3K/Akt cell survival signaling pathway [112].

One of the cardiovascular complications in type 2 diabetes mellitus is diabetic cardiomyopathy which is defined as changes in morphology and function of the myocardial leading to cardiac dysfunction and eventually heart failure [113]. Activation of cell death pathways is reported as one of the pathophysiologies of diabetic cardiomyopathy [114]. Li et al. investigated the effects of paeonol on diabetic cardiomyopathy [115]. The result revealed that rats treated with paeonol attenuated apoptosis of myocardial tissues followed by improvement of cardiac function and myocardial morphology compared to the diabetes group. In addition, paeonol treated group showed decreased glucose serum level, ROS, inflammatory mediators (TNF-α, IL-6, and IL-1β), and reduced collagen deposition in the cardiac tissue. The cardioprotective effect of paeonol was achieved via modulation of PI3K, p-Akt, glycogen synthase kinase-3β, and glycogen synthase followed by down-regulation of apoptotic and inflammatory signaling protein such as protease-activated receptor-1, caspase-3, TNF-α, NF-κB p65, and p-Iκ-Bα expressions. The result suggests that paeonol protects against diabetic cardiomyopathy and improves myocardial function by reducing apoptosis, inflammation, and activation of the PI3K/Akt-GSK-3β signaling pathway [115].

The effects of paeonol against cardiovascular diseases and the related mechanisms are discussed in the subsections below (Table 1).

## 4. Conclusions

In conclusion, paeonol, a naturally occurring bioactive compound in Cortex Moutan, has been shown to be a promising therapeutic agent for the treatment of cardiovascular diseases. These cardioprotective effects are attributed to its multifactorial actions such as anti-inflammatory, anti-oxidant, anti-apoptotic, and regulation of vascular tone. Paeonol’s pleiotropic pharmacological effects suggest that it has a lot of potential for clinical use in atherosclerosis prevention and treatment. In terms of paeonol’s undesirable physical properties, there have been reports that several paeonol-loaded carriers have overcome the shortcomings of poor solubility and stability, improving the bioavailability and residence of paeonol in vivo, and thus providing reliable technical support for paeonol in practice. However, scientific investigations examining paeonol’s therapeutic effects have been uncommon in recent years. To assess the efficacy and safety of the treatment, large randomized, controlled, and double-blind trials are urgently needed.

## Figures and Tables

**Table 1:** Mechanism of action of Paeonol in cardiovascular diseases.

Effects	Models	Mechanism	Reference
Anti-oxidative effect	Oxidized low-density lipoprotein induced HUVEC apoptosis	↓ LOX-1, ↓ P38 MAPK, ↓ NF-κB, and ↓ Caspase-3	[48]
	Paeonol protects against premature senescence in HUVEC cells	↓ P53, ↓ Ac-H3 K14, ↓ Ac-H4 K16 and ↓ SA-beta-galactosidase ↑ Sirt1, ↑ BrdU and ↑ Cell growth profile	[50]
	Paeonol attenuated intracellular lipid accumulation in RAW264.7 macrophages and ApoE^−/−^ mice	↓ HO-1, ↓ CD36, ↓ c-Jun-AP-1, and ↓ Calpain ↑ ABCA1 and ↑ Wogonin	[51]
Anti-inflammatory	Rat aortic endothelial cells (RAECs).	↓ TNF-α-induced ↓ monocytic cell adhesion to rat aortic endothelial cells by of VCAM-1 expression ↓ VCAM1 viaregulation of ERK ½ and p38	[58]
	Rat model of carrageenan-evoked thermal hyperalgesia, bFGF stimulated HUVECs	↓ TNFα, ↓ IL-1ß, ↓ iNOS, ↓ COX-2 and ↓ PGE2 ↓ essential angiogenesis pathways such as proliferation and migration in FGF	[59]
	Ox-LDL stimulated VECs isolated from rat thoracic aorta	↑ miR 126 expression to monocyte adhesion ↓ PI3K/Akt/NF-κB signalling pathway	[60]
	Ox-LDL stimulated VECs isolated from rat thoracic aorta	↑ survival rate of ox-LDL treated VECs ↓ Release of ox-LDL induced TNF-α reverse the PTEN expression	[61]
	High fat diet ApoE^−/−^ mice & HUVECs	↑ miR 223 expression ↓ STAT3 pathway ↓ IL-1ß and IL6 ↓ VCAM 1 and ICAM 1	[62]
	Wistar rats with ischemic reperfusion (IR) injury	↓ No-reflow area ↓ myocardial ischemic damage ↓ CK, CRP, LDH, TnT levels	[63]
	ApoE^−/−^ mice	↑ LXRα-ABCA1–dependent cholesterol efflux	[64]
Regulation of vascular tone	Spontaneously Hypertensive rats	↓ elevated blood pressure and increased the cerebral blood flow velocity ↓ vascular endothelium injury	[83]
	Isolated SD rat aorta preparation	NO dependent vasodilator effects (-) voltage-dependent and receptor-operated Ca^2+^ channel, as well as intracellular Ca^2+^ release	[13,76]
	Tunicamycin-induced endothelial dysfunction in mice and HUVECs	↑ AMPK, ↑ PPARγ, ↑ peNOS and ↑NO ↓ AT6, ↓ GRP78, ↓ peIF2α and ↓ ROS,	[78,79]
	LPS-induced endothelial dysfunction in mice and HUVECs	↓ TLR4, ↓ BMP4, ↓ ROS, ↓ p38 and ↓ iNOS, ↑ peNOS and ↑ NO	[82]
Anti-apoptosis effect	Epirubicin-induced Cardiotoxicity in rat cardiac myocytes H9C2 and mice cardiomyocytes LH-1 cell death	↓ Caspase 3, ↓ Bax and ↓ PI3K/AKT/mTOR ↑ Bcl-2	[104]
	I/R-induced apoptosis in H9c2 embryonic rat myocardium-derived cells	↓ Caspase 3, Bax ↑ Bcl-2, Notch1	[110]
	(ISO)-induced myocardial infarction in rats	↓ Fas, ↓ TNF-α, ↓ Bax, ↓ caspase-3, ↓ caspase-8, ↓ caspase-9, ↓ cytochrome c, ↓ CK-MB, ↓ cTnI and ↓ cTnT ↑ Bcl-2/Nrf2/PI3K/Akt	[112]
	Streptozotocin-induced diabetic cardiomyopathy model in rat	↓ caspase-3, ↓ TNF-α, ↓ IL-6, ↓ NF-κB, ↓ p65 and ↓ p-Iκ-Bα ↑ PI3K/Akt-GSK-3β	[115]

## Data Availability

Not applicable.

## Abbreviations

Ac-H3 K14	histone H3 acetylation at lysine 14
Ac-H4 K16	histone H4 acetylation at lysine 16
AIF	apoptosis inducing factor
ApoE^−/−^	apoliprotein E-deficient
BrdU	Bromodeoxyuridine/5-bromo-2’-deoxyuridine
CD36	cluster of differentiation 36
c-Jun-AP-1	c-JUN-Activator protein-1
CK	creatinine kinase
CK-MB	creatine kinase-MB
EDCF	endothelium-derived contracting factors
EDHF	endothelium-derived hyperpolarizing factor
EDRFs	endothelium-derived relaxing factors
eNOS	endothelial nitric oxide synthase
HUVECs	human umbilical vascular endothelial cells
LOX-1	lipoprotein receptor-1
LTT assay	lymphocyte transformation test assay
LVEDD	left ventricular end-diastolic dimension
MCP-1	monocyte chemoattractant protein-1
PRRs	pattern recognition receptors
RAECs	rat aortic endothelial cells
SA-beta-galactosidase	Senescence-associated beta-galactosidase
sGC	soluble guanylyl cyclase
SIRT 1	Sirtuin 1
STZ	streptozotocin
TUNEL	deoxynucleotidyl transferase-mediated dUTP nick-end labelling
VECs	vascular endothelial cells
